# Rapid reduction of nitroarenes photocatalyzed by an innovative Mn_3_O_4_/α-Ag_2_WO_4_ nanoparticles

**DOI:** 10.1038/s41598-020-78542-5

**Published:** 2020-12-09

**Authors:** Mohamed Mokhtar Mohamed, Hassan El-Farsy

**Affiliations:** 1 Chemistry Department, Faculty of Science, Benha University, Benha, Egypt; 2 Chemistry Department, Faculty of Science, Al-Azhar University, Nasr City, Cairo, Egypt

**Keywords:** Environmental sciences, Energy science and technology

## Abstract

A novel photocatalyst based on the design of P-N heterojunction between hollow spherical Mn_3_O_4_ and nanorods shape of α-Ag_2_WO_4_ is synthesized using a sonication-deposition–precipitation route. The nanocomposite Mn_3_O_4_/α-Ag_2_WO_4_(60%) exhibits a great potential towards nitroarenes (including 4-nitrophenol, 4-nitro-aniline and 4-Nitro-acetanilide) reduction under visible light irradiation exceeding that of Mn_3_O_4_/α-Ag_2_WO_4_(40%) as well as their individual counterparts (3–5%). The Mn_3_O_4_/α-Ag_2_WO_4_(60%) catalyst exhibited an excellent photo-reduction activity comprised of 0.067 s^−1^ towards 4-nitrophenol (0.001 M) in only 60 s reaction time using NaBH_4_ (0.2 M). This was due to the successful formation of the Mn_3_O_4_/α-Ag_2_WO_4_ composite as validated by XRD, TEM-SAED, XPS, FTIR, UV–Vis diffuse reflectance and PL techniques. Decreasing the E_g_ value into 2.7 eV, the existence of a new (151) plane in the composite beside enhancement of the composite electrical conductivity (1.66 × 10^–7^ Ω^−1^ cm^−1^) helps the facile nitroarenes adsorption and hydrogenation. Transient photocurrent response and linear sweep voltammetry results prove the facilitation of photogenerated charge carriers separation and transport via improving electron lifetime and lessening recombination rate. The composite photocatalyst produced higher amounts of H_2_ production, when inserted in a typical reaction medium containing NaBH_4_, comprised of 470 µ mole/g exceeding those of the counterparts (35 µ mole/g). This photocatalyst is strikingly hydrogenated 4-nitrophenol under mild conditions (25 °C and 0.35 MPa pressure of H_2_) with magnificent rate constant equal 34.9 × 10^−3^ min^−1^ with 100% selectivity towards 4-aminophenol.

## Introduction

Silver tungstate (Ag_2_WO_4_) of different structural phases, wide band gap (E_g_ ≥ 2.9 eV) and low surface area has shown a remarkable activity as biocide material and photocatalyst in the degradation of different pollutants under UV irradiation^1–4^. This enhanced photoactivity is mainly attributed to the approved photosensitizing capability, distinctive crystalline features, and to the throughput of crystal defects^5^. However, the limited light absorption margin of Ag_2_WO_4_ is principally due to the large band gap and to the fast recombination of charger carriers. Ag_2_WO_4_ suffers instability via inducing photocorrosion during prolonged light illumination^6^ as a result of Ag^+^ dislodgment into Ag; during reusing and thus inhibits the visible light absorptivity of the Ag_2_WO_4_ photocatalyst. Accordingly, coupling Ag_2_WO_4_ with other semiconductors such as ZnO and/or Fe_3_O_4_^7,8^ or through the addition of plasmonic metals such as Ag^9–12^ or forming a heterojunction with g-C_3_N_4_^13^ has shown greater photocatalytic activity towards degradation of some organic compounds; including methylene blue and rhodamine-B, compared to individual analogues under visible light illumination. Lately, a surface plasmon catalyst composed of Ag_2_WO_4_/Ag/Bi_2_MoO_6_ reported by Lv et al.^14^ indicates a significant activity in degradation of methylene blue under visible-light irradiation. However, the mentioned nanocomposite has shown some drawbacks such as taking very long time to accomplish the degradation, evolution of different Ag_2_WO_4_ structures of varied crystallinity and PL emissions, fast recombination of electron/hole and photocorrosion enhancement thru prolonging the illumination time^5–8,13–17^.

Hausmannite Mn_3_O_4_ that displays one of the most stable crystal structures of Mn_x_O_y_ has brought significant research attention, wherein Mn^2+^ and Mn^3+^ are respectively in tetrahedral and octahedral positions. It also shows distinctive structural features along with wide potential applications^18–20^. Inspired by the p-type semiconducting property of Mn_3_O_4_ and its large potential in boosting oxygen production when involved in nanocomposites formation, it shows unique photocatalytic characteristics. This primarily based on the evolution of highly reactive oxygen species^21–23^. Based on different Mn_3_O_4_ morphological structures processed via different routes in the presence of ZnO, graphene and Fe_3_O_4_/graphene, an appreciable visible light oxidative degradation property for different organic pollutants was achieved^7,24–26^. The respects and recognition of the serious demand to discover families of new materials other than the well-studied oxide semiconductors such as TiO_2_ and WO_3_ have achieved considerable momentum recently. Accordingly, significant attempts have been dedicated to the shift of the V_B_ and C_B_ edge locations of different semiconductors (“bandgap construction”) to modify their interfacial energetics to the targeted photooxidation or photoreduction processes.

Apparently, neither Ag_2_WO_4_ nor Mn_3_O_4_ is employed in nitroarenes photoreduction unlike metal oxides supported noble metals^27–31^, which use to suffer a rapid decrease in the activity due to metal aggregation and leachability in addition to the noble metal high prices that limits its usage on a large-scale.

Based on the above knowledge, there are yet no articles devoted on the combination of Mn_3_O_4_ with Ag_2_WO_4_ to configure their impact on the photocatalytic reduction of nitroarenes under visible light illumination. The heterojunction based on Ag_2_WO_4_/Mn_3_O_4_ is expected to possess an excellent candidate capable of nullifying the photocorrosion of Ag_2_WO_4_; based on the electron transfer from Mn^4+^/Mn^3+^ and Mn^3+^/Mn^2+^ moieties manipulated by structural changes^30,31^, amplify light harvesting as well as expanding the charges separation. In the present study, we have successfully utilized ultrasonication-deposition–precipitation route to fabricate Mn_3_O_4_/Ag_2_WO_4_ nano-composite in the absence of any stabilizing agents with no agglomeration and high performance towards nitroarenes photo-reduction. From the practical point of view we used also molecular hydrogen as the cleanest and environmental friendly reducing agent for 4-NP and at room temperature to obviate energy consumption and suspicious safety problems. The morphology, crystal structure, surface properties and optical properties of the nano-composites and individual analogues have been thoroughly studied.

## Results and discussion

### Bulk, morphology and elemental composition

**Figure 1:**
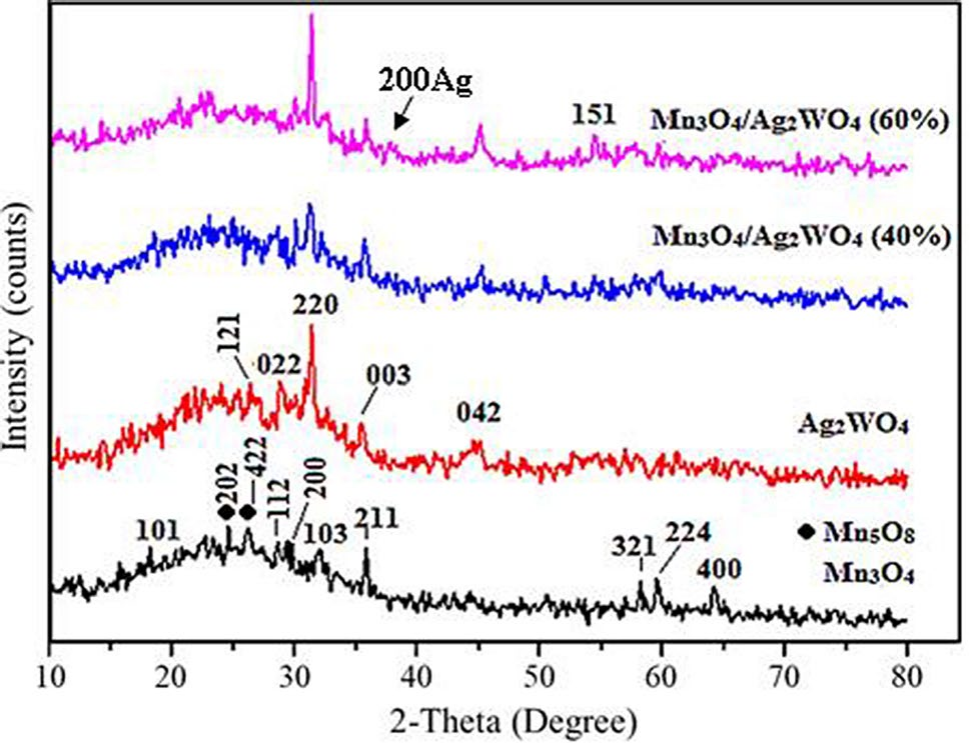
XRD patterns of Mn_3_O_4_, α-Ag_2_WO_4_, Mn_3_O_4_/α-Ag_2_WO_4_ (40%) and Mn_3_O_4_/α-Ag_2_WO_4_ (60%).

The XRD spectra of pure Mn_3_O_4_ and Ag_2_WO_4_ catalysts together with the nanocomposites of Mn_3_O_4_/Ag_2_WO_4_ are shown in Fig. 1. The Mn_3_O_4_ peaks positioned at 2θ equal 18.1°, 28.9°, 30°, 33°, 36.1°, 58.4°, 60° and 64.5° are respectively designated to (101), (112), (200), (103), (211), (321), (224) and (400) crystal planes of hausmannite tetragonal Mn_3_O_4_ (JCPDS card: 89–4837). Whilst the remaining peaks perceived at 24.5° and 26.2° are correspond to (202) and (422) planes of Mn_5_O_8_ (16,956-ICSD), conceivably resulted from further oxidation of Mn_3_O_4_. The spectrum of Ag_2_WO_4_ shows prominent peaks at 2θ values 26.63°, 29°, 31.56°, 32.71° and 44.9° correspond respectively to the crystal planes (121), (022), (220), (003) and (042) of orthorhombic *α*-Ag_2_WO_4_ nanostructures (JCPDS No.: 34-0061).

The XRD patterns of the Mn_3_O_4_/Ag_2_WO_4_ composites were originated from the mixed phases of tetragonal Mn_3_O_4_ and orthorhombic *α*-Ag_2_WO_4_. The two phase composition of Mn_3_O_4_ and Ag_2_WO_4_ in the nanocomposites have shown significant decrease in peaks intensities at the 40% Ag_2_WO_4_ loading. On the other hand, a tremendous increase is obtained for the (220) plane of Ag_2_WO_4_ at the 60% loading. Nonetheless, a new phase for Ag_2_WO_4_ never obtained in its pure form existed at 2θ = 54.6° is depicted and ascribed to the (151) plane, developed from the evolution of the Ag_2_WO_4_ hexagonal structure^7,24^. The measured crystallites size determined using the Scherrer’s equation indicate average sizes of 28 nm and 34 nm for the Mn_3_O_4_ and Ag_2_WO_4_, respectively. Whereas, the average crystallite size was 21 nm for Mn_3_O_4_/Ag_2_WO_4_ (40%) and 53 nm for Mn_3_O_4_/Ag_2_WO_4_ (60%), which exhibits larger d-spacing and minor shifts in peak positions to lower angles than the former. This provokes that a unit cell expansion is developed in Mn_3_O_4_/Ag_2_WO_4_ (60%) with a strong strain owing to planar stresses resulted from stoichiometry alteration. Moreover, larger sizes could also cause such shifts to lower XRD angles.

**Figure 2:**
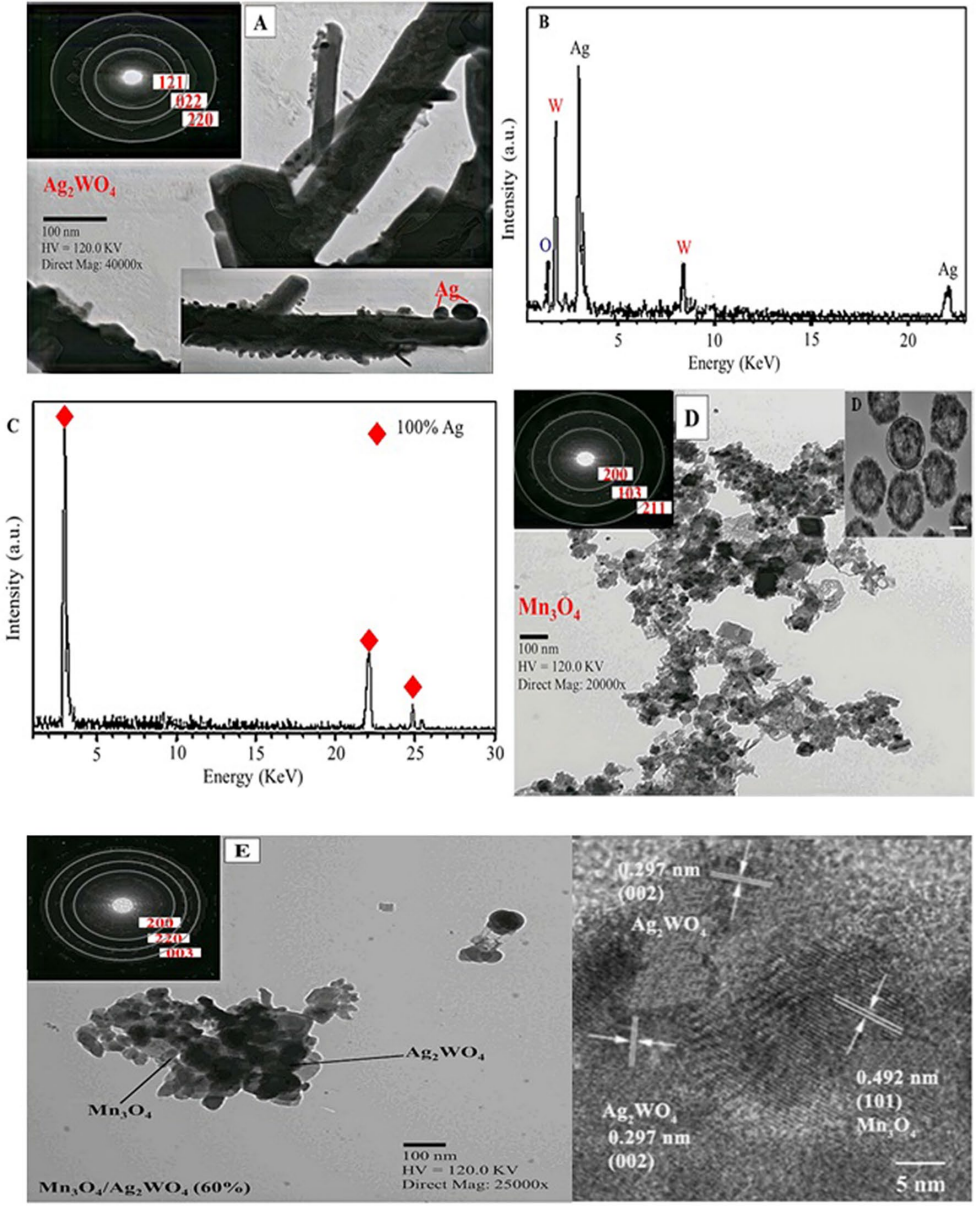
TEM-SAED (inset) images of **A**) α-Ag_2_WO_4_, (**B**) EDS spectrum of α-Ag_2_WO_4_, (**C**) EDS spectrum of Ag nanoparticles deposited on α-Ag_2_WO_4_, (**D**) TEM of Mn_3_O_4_ and the inset is the HRTEM of hollow Mn_3_O_4_, (**E**) Mn_3_O_4_/α-Ag_2_WO_4_ (60%) catalysts and HRTEM image; the scale bar represents 100 nm in all images.

The morphological structure of *α*-Ag_2_WO_4_ analyzed by the TEM-SAED measurements; and shown in Fig. 2A, discloses the existence of nanorods shape of an average diameter of 37 nm and few micrometer length. A deposition of Ag nanospherical particles; of an average diameter of 18 nm, on the Ag_2_WO_4_ nanorods was depicted although it is never seen by XRD results. The inset Figure associated to the selected area electron diffraction of *α*-Ag_2_WO_4_ indicates few spots of diffraction allocated on concentric spheres. They are consistent with the planes of (121), (022) and (220) of Ag_2_WO_4_ those were in harmony with its XRD pattern, depicted in Fig. 1. The EDS operated on Ag_2_WO_4_ (Fig. 2B) accomplished its absolute existence via presence of the elements Ag, W and O beside the predominance of Ag atoms of spherical character deposited on the former nanorods. The EDS analysis of the nanospheres (marked by black circles in the in-situ Fig. 2A) shows only the existence of Ag nanoparticles (100 at % Ag) point to the formation of Ag on the surface of Ag_2_WO_4_; as shown in Fig. 2C.

Figure 2D shows the image of hollow spherical-like shape of Mn_3_O_4_ with average diameters of ~ 30 nm. The inset shows ring patterns of uniform structure with the plane lines (200), (103) and (211) correspond to Mn_3_O_4_. Figure 2E shows the image of Mn_3_O_4_/Ag_2_WO_4_ (60%) that hold opposing shapes compared to the individual analogue. The Ag_2_WO_4_ image in the composite resembles spheroidal-like structure whereas Mn_3_O_4_ configures as rice beads wherein the former coating that of the latter. The particle diameter of the composite is in the span range of 25–35 nm. The SAED pattern reveals the presence of clear fringes with different spacing's correspond to the (200) plane of Mn_3_O_4_ and (220), (003) planes of Ag_2_WO_4_, those were in excellent conformity with the XRD results. Changing the morphology of the composite from those of individual correspondents confirms the strong attachment between the two structures and rather depicts the successful formation of the Mn_3_O_4_/Ag_2_WO_4_ composite.

**Figure 3:**
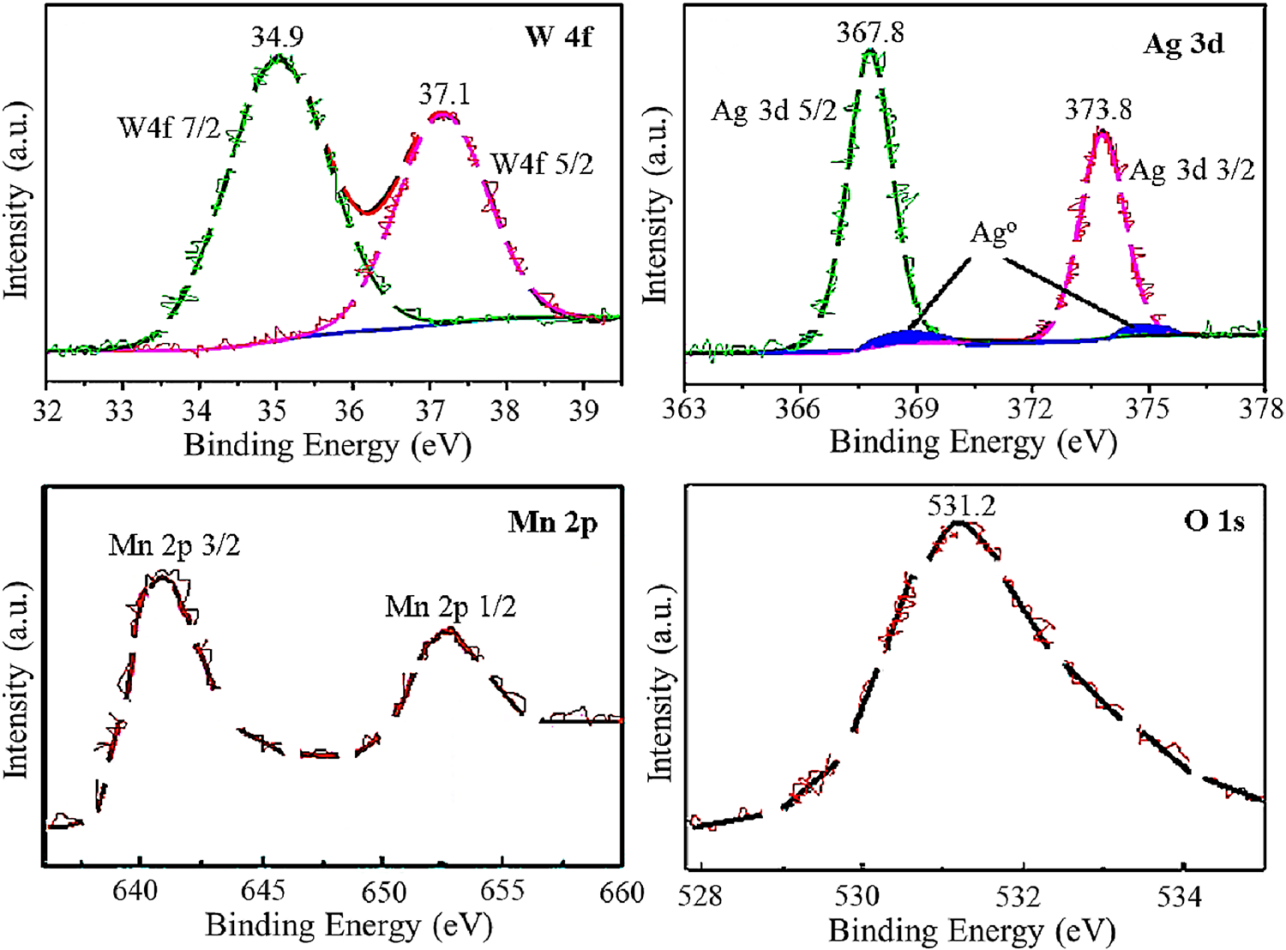
X-ray photoelectron spectra of W 4f., Ag 3d, Mn 2p, and O 1 s of 15 wt% Mn_3_O_4_/ Ag_2_WO_4_ (60) catalyst.

The XPS technique was employed to further justify the surface compositions of the Mn_3_O_4_/Ag_2_WO_4_ (60%) catalyst (Fig. 3). The W4f_7/2_ and 4f_5/2_ peaks detected at 34.9 and 37.1 eV are typically correlated to W^4+^ tungstate oxide structure^32^. The Ag3d_5/2_ and 3d_3/2_ detected at 367.8 and 373.8 eV revealed the presence of Ag^+^, comparable to those similarly seen in other oxide structures^33^.

Whereas, the small broad blue shaded peaks depicted at 368.8 eV and 375.1 eV implied the existence of Ag°^27^. The binding energies noticed at 642.0 eV and 653.2 are attributed to the existence of Mn^3+^ of Mn_3_O_4_^34^. The O1s peak positioned at 531.2 eV is consistent with the lattice oxygen coped with those observed in both Mn_3_O_4_ and Ag_2_WO_4_ structures^33,34^. Hence, the structure of the Mn_3_O_4_/Ag_2_WO_4_ (60%) nanocomposite is clearly identified and the amount of Mn is verified at 39.4 wt% whereas those of Ag^o^ is quantitatively exhibited a value of 2%, to finally propose the existence of Ag^o^(2%)/Mn_3_O_4_(40%)/Ag_2_WO_4_(60%) elements in the composite.

### Vibrational, electronic and conductivity characteristics

**Figure 4:**
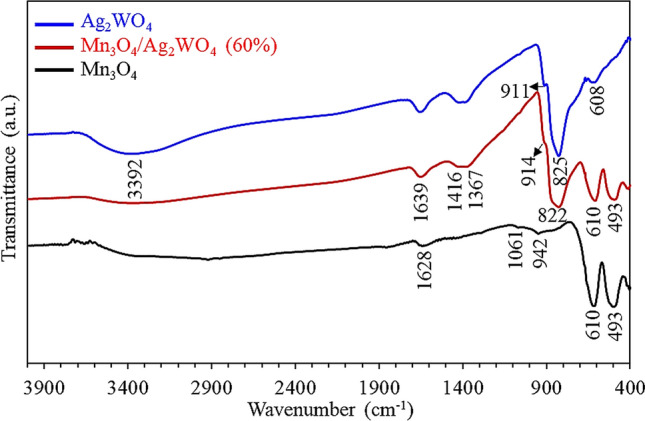
FTIR spectra of α-Ag_2_WO_4_, Mn_3_O_4_ and Mn_3_O_4_/α-Ag_2_WO_4_ (60%).

To have an idea about the functional groups verified on the as-synthesized catalysts, FT-IR spectroscopic examination was carried out in the wavelength range of 4000–400 cm^−1^ (Fig. 4). The FT-IR spectrum of Mn_3_O_4_ exposes distinctive bands at 493 and 610 cm^−1^ connected to the Mn–O stretching vibration modes in tetrahedral and octahedral positions, respectively^35^.

The weak absorption bands at 942 cm^−1^ and 1061 cm^−1^ are attributed to the C–C bond and CH_2_ rocking in PVP pointing to the presence of template residuals. Whereas, the bands at 1628 cm^−1^ and 3400_broad_ cm^−1^ are related respectively, to water molecules stretching vibrations and hydrated free O–H groups^36^. The FTIR spectrum of Ag_2_WO_4_ indicates a band at 608 cm^−1^; due to the stretching mode of W–O in WO_6_, beside bands at 825 cm^−1^ and 911 cm^−1^ ascribed to O–W–O asymmetric stretching vibration of different structures. The IR modes at 3392 cm^−1^ and 1639 cm^−1^ are assigned respectively to O–H stretching and bending vibrations of adsorbed H_2_O molecules on the catalyst surface. Whereas, those at 1416 and 1367 cm^−1^ are designated to O–H deformation vibrations of tertiary C–OH and bending absorption of the carboxyl group. The spectrum of the Mn_3_O_4_/Ag_2_WO_4_ (60%) composite exhibits intense bands due to Mn_3_O_4_ (including 493_sh_ and 610 cm^−1^) and Ag_2_WO_4_ (comprising 822 and 914 cm^−1^), signifying the successful construction of the mixed phase Mn_3_O_4_/Ag_2_WO_4_. Changing the environment in the composite affects the bond length causing shifts in wavenumber either to higher (914 cm^−1^) or to lower (822 cm^−1^) frequencies; compared to pure counterparts, definitely because electronegativity is expected to involve changes in bond length. Decreasing the intensities of the composite bands when correlated to their pure samples is diagnostic of decreasing the functional groups concentration in the composites. Specifically, bands belonging to Mn–O group in the Mn_3_O_4_/Ag_2_WO_4_ (60%) composite exhibit much weaker intensity than that of pure Mn_3_O_4_, ascribed to the reaction of Mn–O groups of Mn_3_O_4_ with Ag–O and W–O groups of Ag_2_WO_4_. This could cause an intrinsic disorder in the composite beside expected changes in crystal field, dipole and electronic band structures of valence band and conduction band; apart from the individual analogue. This is expected to alter the behaviors of photo-generated charge carriers as well as the excitation processes^30,31^. The shift of the OH stretching band into lower wavenumbers (3388 cm^−1^) besides its broadening is correlated to strengthening of the hydrogen bonding interaction. These results are taken as a criterion for the composite formation.

**Figure 5:**
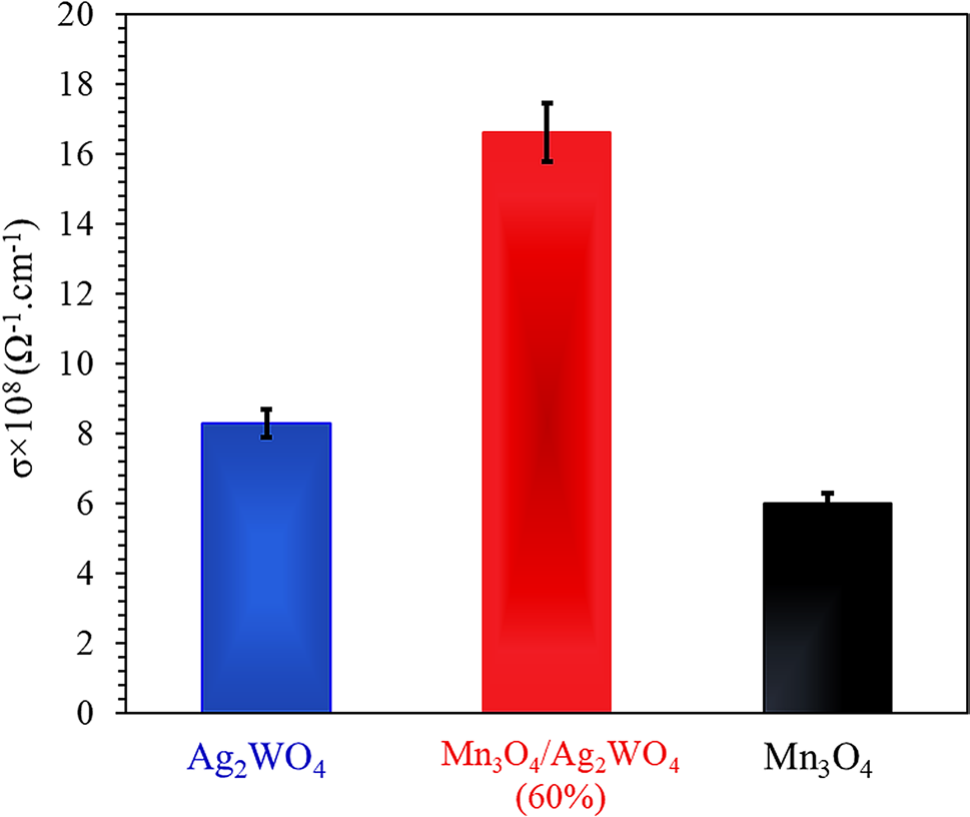
Conductivity measurements of α-Ag_2_WO_4_, Mn_3_O_4_ and Mn_3_O_4_/α-Ag_2_WO_4_ (60%) catalysts.

To prove the mission of electrons during the photoreduction performance, the electrical conductivity of the as-synthesized nanocomposite beside bare Mn_3_O_4_ and Ag_2_WO_4_ catalysts was evaluated at room temperature (Fig. 5). The electrical conductivity values follow the order: Mn_3_O_4_/Ag_2_WO_4_ (60%) [1.66 × 10^–7^ Ω^−1^ cm^−1^] > Ag_2_WO_4_ [8.28 × 10^–8^ Ω^−1^ cm^−1^] > Mn_3_O_4_ [5.98 × 10^–8^ Ω^−1^ cm^−1^]. This indicates that the combination of Ag_2_WO_4_ with Mn_3_O_4_ is effectively amplified the electronic conductivity compared to the pure correspondents. It also signifies the synergism between the latter components and verifies in addition the increase of the hopping rate in spite of the substitution of Ag_2_WO_4_ into Mn_3_O_4_. This could give a clue about the good contact between the components forming the nanocomposite although of exceeding their crystallites size. The superiority of crystallites size of Mn_3_O_4_/Ag_2_WO_4_ (60%) is going to decrease scattering of the free electrons if compared with the smaller crystallized ones based on the fact that conductivity is inversely proportional to the electron scattering γ(σ = N_e_e^2^ = me * γ)^38^. The above results were also well confirmed from the resistance values, which were found to decrease in the sequence; Mn_3_O_4_/Ag_2_WO_4_ (60%) [2.17 × 10^6^ +Ω] < Ag_2_WO_4_ [4.35 × 10^6^ Ω] < Mn_3_O_4_ [9.81 × 10^6^ Ω].

**Figure 6:**
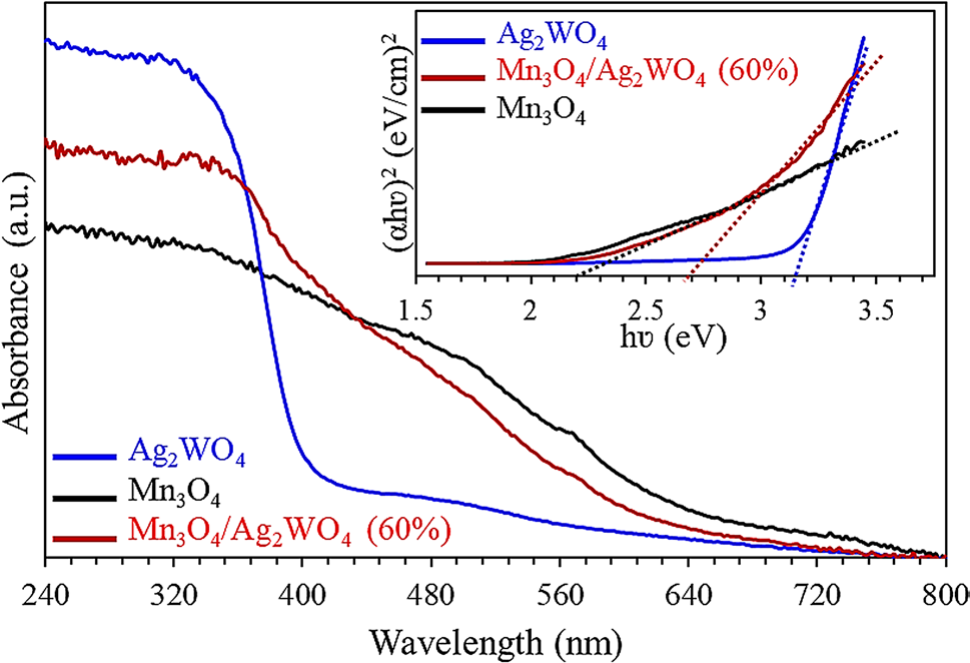
UV–visible spectra of α-Ag_2_WO_4_, Mn_3_O_4_ and Mn_3_O_4_/α-Ag_2_WO_4_ (60%) with the band gap energy plots as insets.

Figure 6 displays UV–Vis absorption spectra of Mn_3_O_4_, Ag_2_WO_4_ and Mn_3_O_4_/Ag_2_WO_4_ (60%) catalysts in the 240–800 nm margin. The Ag_2_WO_4_ catalyst shows the strongest absorption in the UV breadth expanded into the visible light absorption range via exposing an edge at 415 nm. Indeed, it gives the lowest visible light regime in the range from 400 to 800 nm compared to rest of catalysts.

On the contrary, Mn_3_O_4_ exposes the lowest UV absorption regime as well as the highest recorded visible light absorptivity in the 445–800 nm range between all the catalysts. Nevertheless, the nanocomposite Mn_3_O_4_/Ag_2_WO_4_ (60%) catalyst has shown a median behavior throughout the whole range against pure counterparts and in the same time it shows broad small bands at 520 nm and 570 nm typical to those seen on Mn_3_O_4_ (515 nm and 564 nm). Shifting the latter bands into higher frequencies in the nanocomposite spectrum beside its median behavior confirms the composite formation. Besides, it exhibits an increased light harvesting capacity over the range till 440 nm, after which Mn_3_O_4_ took over till the end of the range (800 nm). The band gap energies were evaluated by fitting the absorption data into indirect transition via using the equation *αhν* = *E*_*d*_ (*hν − E*_*g*_)^2^ where E_g_ is the indirect band gap, *E*_*d*_ is a constant,* α* is an optical absorption coefficient and *hν* is the photon energy^39^. In this essence, the E_g_ values were 2.2, 3.15 and 2.7 eV for Mn_3_O_4_, Ag_2_WO_4_ and Mn_3_O_4_/Ag_2_WO_4_ (60%) respectively. Apparently, the E_g_ value of the nanocomposite lies in the midway between pure catalysts elaborating the exhibited interaction between them and the facile charge transfer.

### Photoluminescence study

**Figure 7:**
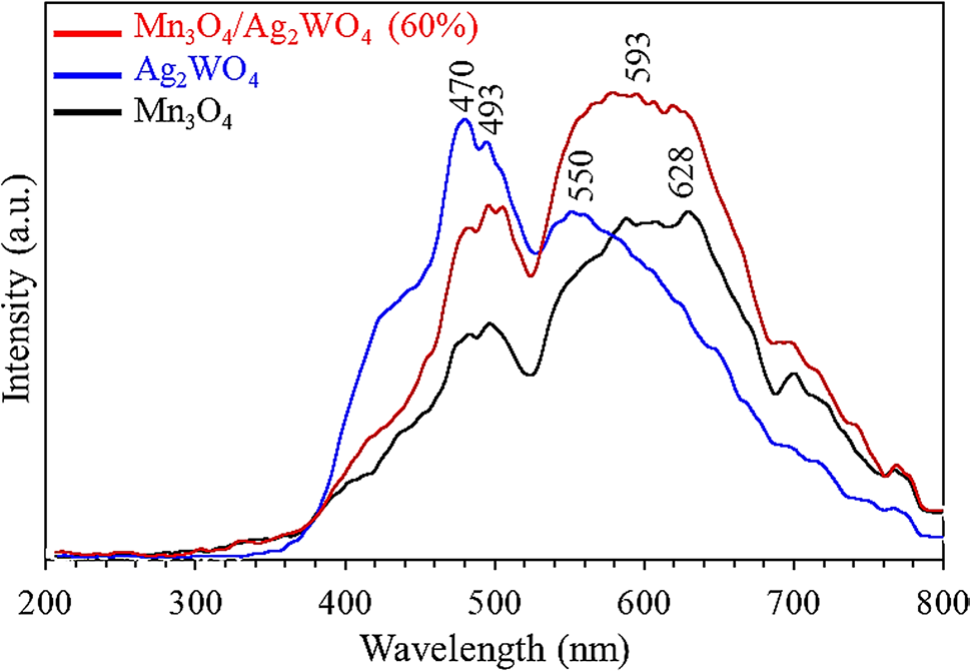
Photoluminescence emission spectra of α-Ag_2_WO_4_, Mn_3_O_4_ and Mn_3_O_4_/α-Ag_2_WO_4_ (60%).

The photoluminescence emission spectra are composed of two broad bands, starting between 440–520 nm and 520–680 nm, and completed at the wavelength of 800 nm. Apparently, the PL first band (440–520 nm) indicates a decrease in PL emission intensities in the order Mn_3_O_4_ < Mn_3_O_4_/Ag_2_WO_4_ (60%) < Ag_2_WO_4_ (Fig. 7), typical to that elaborated in the optical band gap sequence. Apparently, tungstate luminescence is more intense and its maximum emission is localized at higher energy than Mn_3_O_4_ advocating that the electron transfer from oxygen to Mn is easier than that with tungstate cation due to increasing the electronegativity of the latter cation^40^. This behavior was not in a good correlation with the sequence in crystallites diameter decrement. The major emission intensity was for the Ag_2_WO_4_ first band; due to WO_6_ group transition, although the composite spectrum first band shows a red shift compared with pure ones configuring the composite forming effect. Contrarily, the second broad band (yellow emission) of the composite spectrum; maximized at 593 nm, exhibits a major PL emission exceeding those of individual analogues, reflecting high charges recombination within this margin, unlike that of the first one depicted at 510 nm. This is attributed to the d–d transitions engaging Mn^3+^ ions and to the abundant defect. Accordingly, the two different charge transfer transitions committed in the PL emission spectrum of Mn_3_O_4_/Ag_2_WO_4_ (60%) revealed the presence of efficient controlled charge carrier separation (first band-quenched) as well as excessive charges transfer with limited opposition (second band). This implies that Ag_2_WO_4_ can increase or decrease the separation of photo-excited charge transmission of Mn_3_O_4_ depending on the two different combinations exerted between Mn_3_O_4_ and Ag_2_WO_4_.

### Photocatalytic reduction activity of catalysts and their kinetic analyses

**Figure 8:**
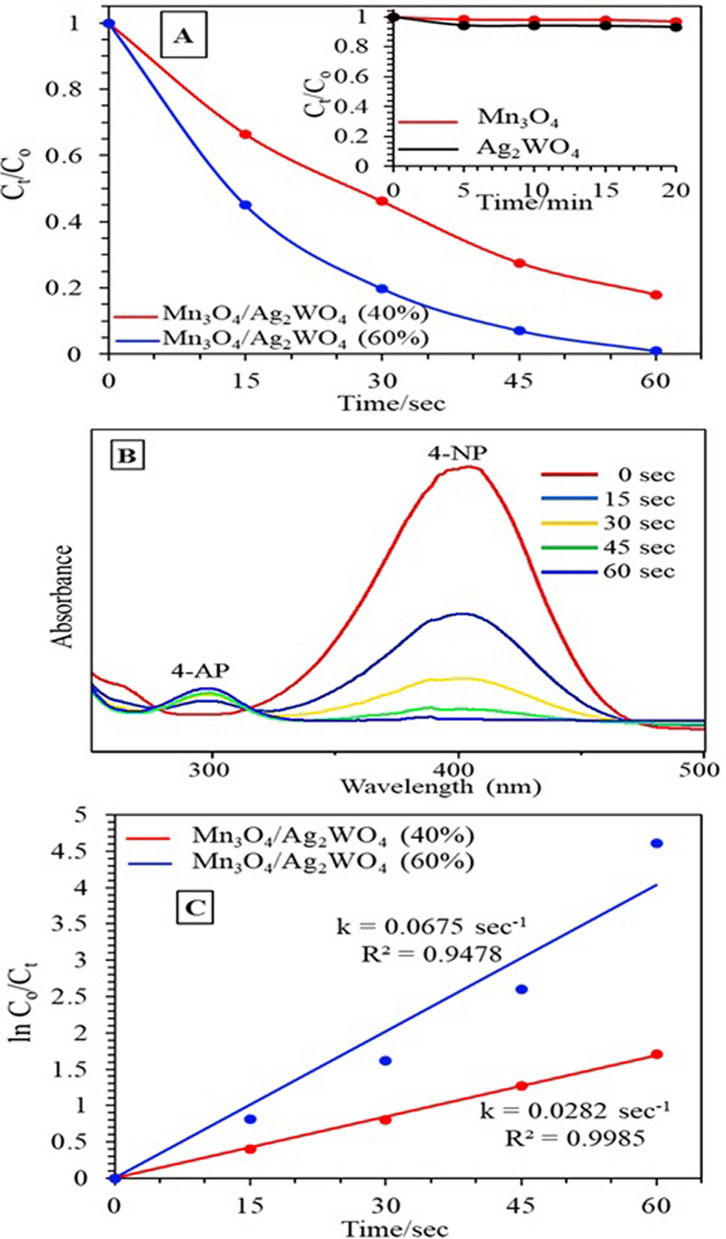
(**A**) The change in the concentration of 4-NP with time on the Mn_3_O_4_/α-Ag_2_WO_4_ (60%) photocatalyst, (**B**) UV–vis absorption spectra of the reduction of 4-NP and (**C**) Plots of Ln (C_t_/C_0_) versus time for the reduction of 4-NP (Reaction conditions: 3 mL 4-NP of conc. 0.001 M, NaBH_4_ conc. of 0.3 M, 3 mg catalyst (1 g/L), T = 289 K, Led lamp 50 W).

The photocatalytic reduction of Mn_3_O_4_, Ag_2_WO_4_, Mn_3_O_4_/Ag_2_WO_4_ (60%) and Mn_3_O_4_/Ag_2_WO_4_ (40%) catalysts is assessed via the reduction of 4-nitrophenol (4-NP) to 4-aminophenol (4-AP) in the presence of NaBH_4_. The plot of C_t_/C_o_ vs. time indicates a sudden decrease in the concentration of 4-NP in the presence of Mn_3_O_4_/ Ag_2_WO_4_ (60%) in only 60 s, in favor of the production of 4-AP at 100% conversion, surpassing Mn_3_O_4_/Ag_2_WO_4_ (40%) that indicates only 80% reduction at the same reaction time (Fig. 8A). Whereas, Mn_3_O_4_ and Ag_2_WO_4_ achieved respectively reduction percentages equal 3% and 5% (inset in Fig. 8A). Control tests performed to validate the photocatalytic characteristic of the reactions and executed under the dark conditions; or without catalyst, indicate no conversion of 4-NP (not shown). Apparently, Fig. 8B shows the characteristic UV–visible spectra of 4-NP (0.001 M) in the presence of Mn_3_O_4_/Ag_2_WO_4_ (60%) and NaBH_4_. The aqueous 4-NP solution that exhibits a maximum at 400 nm after the addition of NaBH_4_; characteristics of nitrophenolate ions, shows a decrease in the deep yellow color that completely bleached in a 60 s reaction time due to the successive 4-AP formation at 300 nm, validated by an isosbestic point. A plot of ln C_t_/C_o_ vs time (s) gives a linear correlation (Fig. 8C) revealing that the reaction displays first-order kinetics with respect to 4-NP with an apparent rate constant of 0.067 s^−1^ and 0.028 s^−1^ for Mn_3_O_4_/Ag_2_WO_4_ (60%) and Mn_3_O_4_/Ag_2_WO_4_ (40%), respectively.

**Figure 9:**
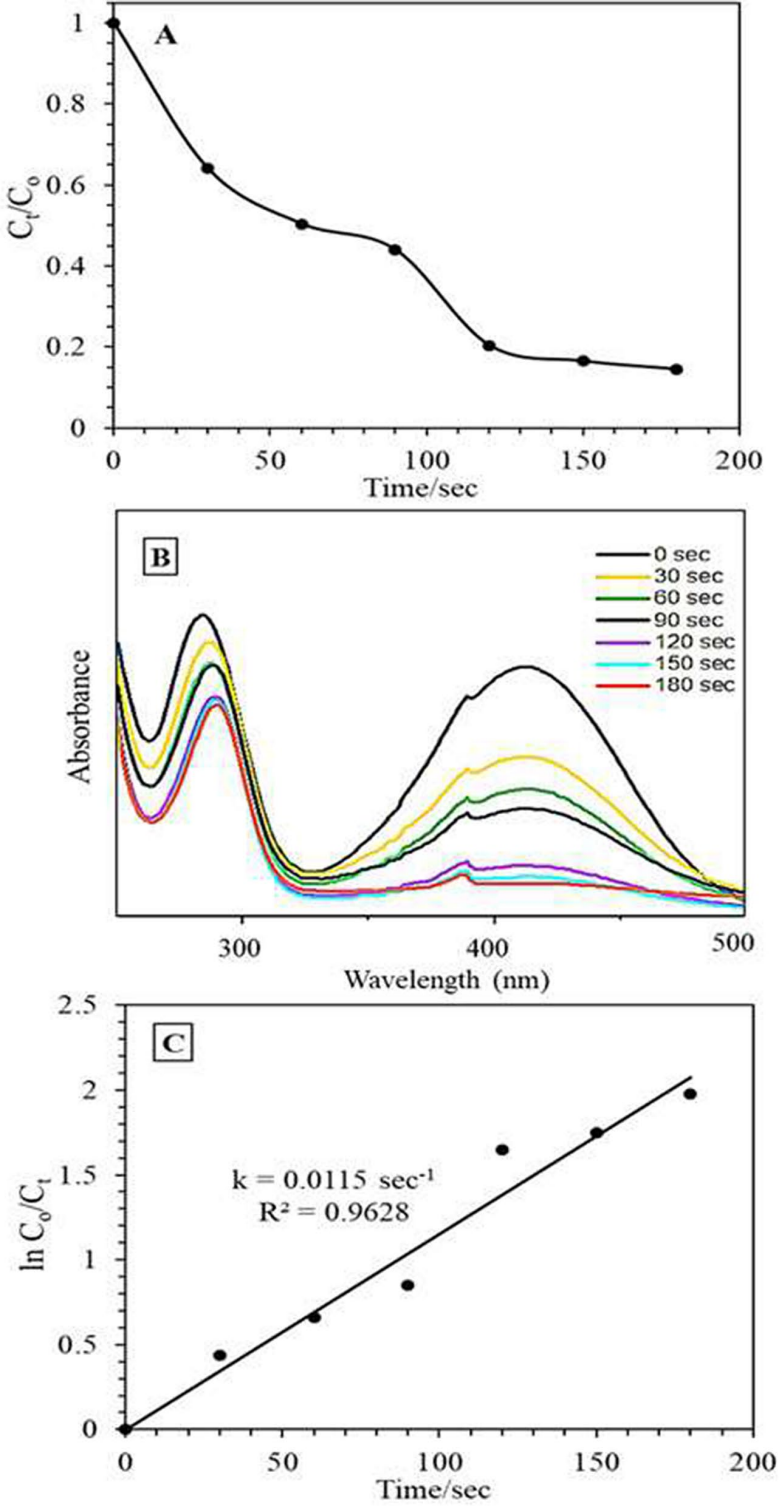
(**A**) The change in the concentration of 4-nitroanilin with time on the Mn_3_O_4_/α-Ag_2_WO_4_ (60%) photocatalyst, (**B**) the change in the concentration of 4-nitroanilin with time, (**C**) Plots of Ln (C_t_/C_0_) vs. time for the reduction of 4-nitroanilin (Reaction conditions: 3 mL 4-nitroanilin of conc. 0.001 M, NaBH_4_ conc. of 0.2 M, 3 mg catalyst (1 g/L), T = 289 K, Led lamp 50 W).

Similarly, the photoreduction of 4-nitro-aniline (4-NA) into 4-amino-aniline (4-AA) on the most active catalyst Mn_3_O_4_/Ag_2_WO_4_ (60%) proceeds to produce 4-AA in 180 s (Fig. 9A, B) with a rate constant of 0.011 min^−1^. The UV–Vis spectra of 4-NA that diminish with time following the addition of NaBH_4_/catalyst demonstrate the developing of 4-AA at 290 nm (Fig. 9C).

The apparent increase in the reaction rate of Mn_3_O_4_/Ag_2_WO_4_ (60%) towards 4-AP than 4-AA by 5 times is probably due to the solvation effect and electron density effect (electron donating effect) by which NH_2_ is expected to increase the polarity; which works as electron rich sites interconnected to a position of conjugation, facilitating the reaction via the formed anions. Exposing the plane (151) on α-Ag_2_WO_4_ when incorporated with Mn_3_O_4_; never seen in the pure form, might increase the reactants absorbability facilitating the reduction consequences.

**Figure 10:**
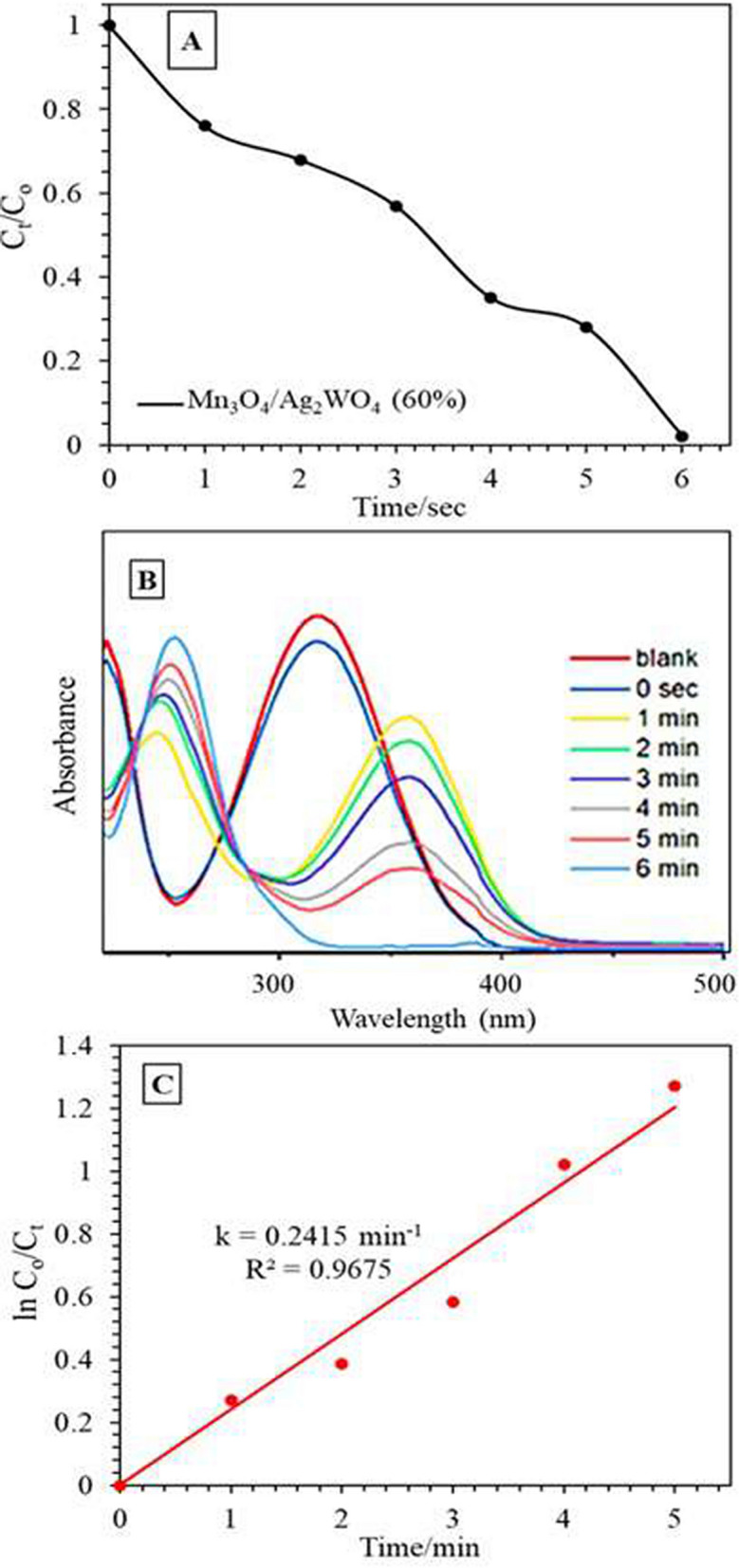
(**A**) The change in the concentration of 4-Nitro acetanilide with time on the Mn_3_O_4_/α-Ag_2_WO_4_ (60%) photocatalyst, (**B**) UV–vis absorption spectra of the reduction of 4-Nitro acetanilide and (**C**) Plots of ln (A_t_/A_0_) versus time. Reaction conditions: 100 mL 4-Nitro acetanilide of conc. 0.001 M, NaBH4 conc. of 0.2 M, 0.1 g catalyst, T = 289 K, Led lamp 50 W.

The comparison of the percentage photoreduction of 4-Nitro acetanilide (4-NAC) to 4-amino acetanilide (4-AAC), calculated from the decrease in the peak at 360 nm of 4-AAC in UV–Vis spectra, as a function of visible light irradiation is shown in Fig. 10A for the Mn_3_O_4_/Ag_2_WO_4_ (60%) catalyst.

After 6 min of visible light exposure, a reduction conversion comprised of 100% was reached. The Langmuir–Hinshelwood (L–H) kinetic model calculated by outlining ln C_o_/C_t_ versus the exposure time (t) as well as C_t_/C_o_ with time indicates a rate constant value of 0.24 min^−1^ (Fig. 10B,C). Apparently, increasing the electronic density of the nanocomposite Mn_3_O_4_/Ag_2_WO_4_ (60%) increases the catalytic photoreduction consequences towards all the nitroarenes, beside the appreciable absorption of light exhibited in the visible light range. Indeed, although the lifecycle of photo-generated electrons is not as high as Mn_3_O_4_; as PL committed, the high electron mobility was the prime reason responsible for enhancing the photo-reduction performance followed by the recombination control of the photo-generated charge carriers.

Increasing the rate constant towards 4-NP photoreduction surpassing those of 4-NA and 4-NAC is greatly dependent on the concentration of nitrophenolate moieties^41^. The activity is well correlated to the reactants solvation. Apparently, hydrogen bonding in nitrophenol is more prominent than in 4-NA and 4-NAC, those possess higher hydrophobicity than 4-NP. Indeed, this hydrophobicity will affect the well dispersion of the catalyst and rather delays attainment of the reactants onto the catalyst active sites. It seems also that the reduction activity has nothing to do with the nitroarenes acidity, which is in the order; 4-NP (pKa = 6.90) < 4-NA (pKa = 1.0) < 4-NAC (pKa = 0.8) indicating that the greater tendency to dissociate protons the stronger is the acid, which specified at lower pKa value. Furthermore, the presence of –C=O–R group in 4-NAC decreases the rate of reaction relative to the H addition. These outcomes signify that Mn_3_O_4_/Ag_2_WO_4_ (60%) offers the best active sites for both NaBH_4_ and 4-NP and exhibits the greatest catalytic activity in contrast to Mn_3_O_4_/Ag_2_WO_4_ (40%) and counterpart catalysts.

### Hydrogenation performance

The catalytic performances of Mn_3_O_4_, Ag_2_WO_4_, Mn_3_O_4_/Ag_2_WO_4_ (40%) and Mn_3_O_4_/Ag_2_WO_4_ (60%) for 4-NP hydrogenation are examined, and the results are elaborated in Table 1. Apparently, the rate constants of the hydrogenation reaction on individual catalysts during one hour reaction time are 2.4 × 10^–3^ and 2.7 × 10^–3^ min^−1^ for Mn_3_O_4_ and Ag_2_WO_4_, respectively at conversion rate in the margin of 8–10%.

**Table 1:** Experimental results of photocatalytic hydrogenation of nitrophenol using the as-synthesized catalysts.

Catalyst name	Conversion (%)	Selectivity (%)	Rate constant (10^–3^ min^−1^)
Mn_3_O_4_	8	100	2.4
Ag_2_WO_4_	10	100	2.7
Mn_3_O_4_/Ag_2_WO_4_ (40%)	71	100	11.8
Mn_3_O_4_/Ag_2_WO_4_ (60%)	98	100	34.9

However, the Mn_3_O_4_/Ag_2_WO_4_ (60%) composite exhibits an excellent catalytic activity under the same conditions via giving a conversion exceeding 98%, and a rate constant as high as 34.9 × 10^−3^ min^−1^.

Evidently, the latter catalyst rate exceeds that of Mn_3_O_4_/Ag_2_WO_4_ (40%) by ~ 3 times. Under dark conditions, the activity of Mn_3_O_4_/Ag_2_WO_4_ (60%) was only 2.35 × 10^−3^ min^−1^ that was far inferior to the reaction rate motivated by light. Hence, visible-light illumination plays the principal role in nitrophenol hydrogenation. Obtaining 100% aminophenol at such mild conditions of low hydrogen amount and at room temperature advocates the photocatalytic efficiency of the Mn_3_O_4_/Ag_2_WO_4_ (60%) catalyst to obtain a good yield. Performing the reaction in water as a solvent instead of ethanol causes a tremendous decrease in the activity into 10.2 × 10^–3^ min^−1^. This is because of lowering the solubility of 4-NP in water as well as the competing of water molecules via their adsorption on the active sits of the catalyst surface.

Based on previous studies and our experimental results, H_2_ and 4-NP will be adsorbed onto the Mn_3_O_4_/Ag_2_WO_4_ (60%) surface and get activated according to the Langmuir–Hinshelwood reaction mechanism. Meanwhile, excited electrons of Mn_3_O_4_ under visible illumination are transferred to the surface of Ag_2_WO_4_, which can further promote the adsorption and activation. With the assistance of electrons as well as the hydrogen activated catalyst, the N=O bond of 4-NP is hydrogenated to form 4-AP. The synergistic effect of the components forming Mn_3_O_4_/Ag_2_WO_4_ (60%) exploits vital role in the photocatalytic reduction of 4-NP via using electrons and hydrogen, wherein Mn_3_O_4_ acts as electron supplier and Ag_2_WO_4_ as active centers. We reached into this hypothesis because in the absence of hydrogen, an appreciable reduction performance was achieved (14.3 × 10^−3^ min^−1^), explaining the role of excited electrons in regulating the reduction process.

### pH effect and recyclability

**Figure 11:**
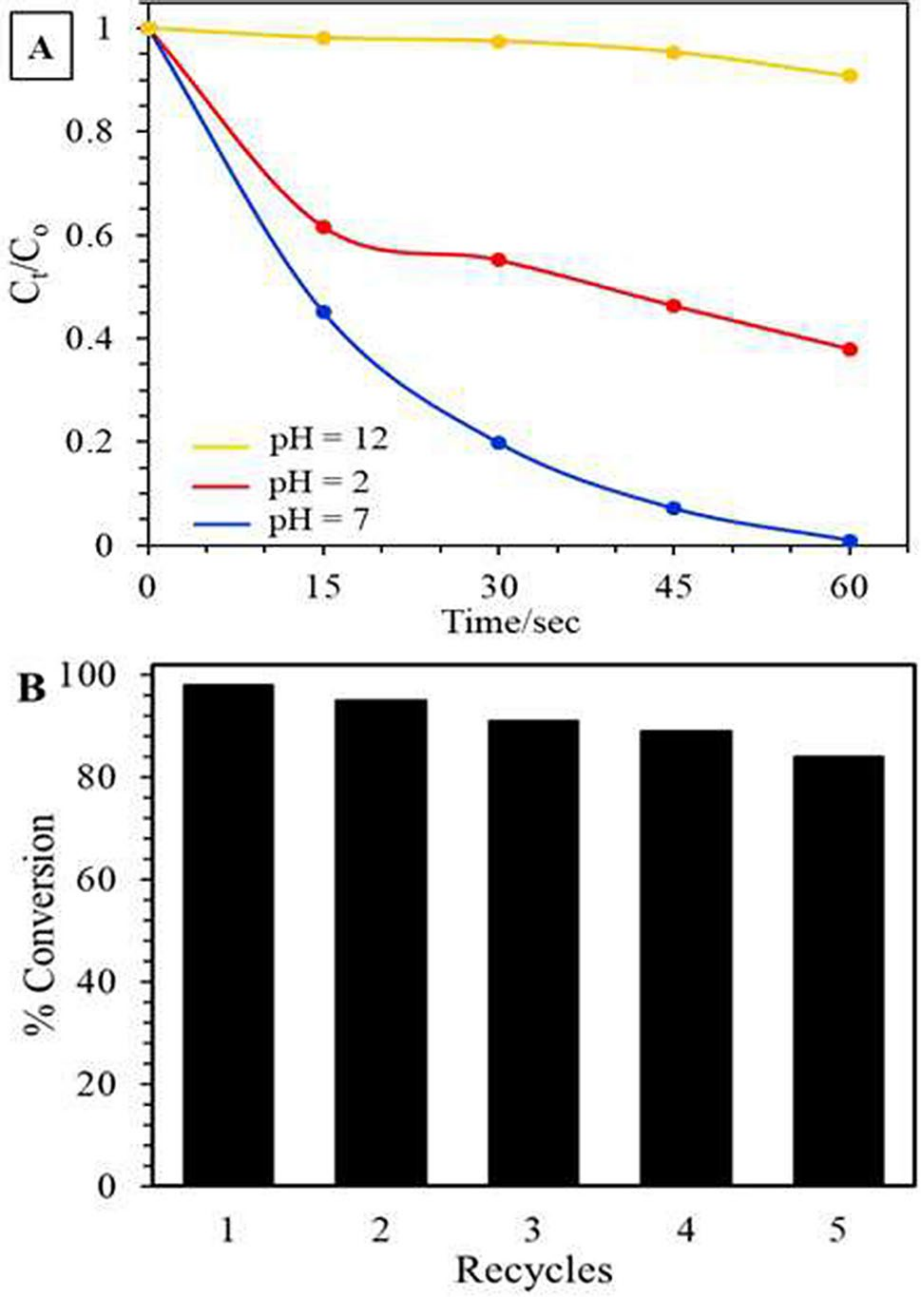
(**A**) The change in the concentration of 4-NP with time in the reduction of 4-NP by Mn_3_O_4_/Ag_2_WO_4_ (60%) at different pH and (**B**) Repeated cycles up to 5 times illustrating the reduction of 4-NP over the same photocatalyst. Reaction conditions: 100 mL 4-NP of conc. 0.001 M, NaBH_4_ conc. of 0.2 M, 0.1 g catalyst.

Figure 11 displays the pH influence on the catalytic activity of Mn_3_O_4_/Ag_2_WO_4_ (60%) towards 4-NP photoreduction. The photoreduction of 4-NP on Mn_3_O_4_/Ag_2_WO_4_ (60%) is greatly dependent on the pH value; median pH is advantageous to an efficient magnificent reduction. A complete reduction of 4-NP is observed within only 60 s at pH equal 7 reflecting the highest K_obs_ of 0.0675 s^−1^. On the other hand, at pH 2 and 12 the photoreduction percentages reach 60% and 5%, respectively. This indeed is relied on the correlation between PZC of Mn_3_O_4_/Ag_2_WO_4_ (60%) and pKa of 4-NP. The measured zero-point charge of Mn_3_O_4_/Ag_2_WO_4_ (60%) was found at pH 7.9 (not shown**)**, meaning that the catalyst surface is positively charged at pH < 7.9. Admittedly, 4-NP photoreduction executed over Mn_3_O_4_/Ag_2_WO_4_ (60%) together with the existence of NaBH_4_ obeyed Langmuir–Hinshelwood kinetics and adsorption is the leading action in the reduction process. Thus, acquiring + ve charge on the nanocatalyst surface facilitates the adsorption of the negatively charged BH_4_^−^, causing an enhanced reduction conversion and superior rate constant of 4-NP photoreduction, under neutral condition. On the other hand, acquiring negative charge on the catalyst surface at pH equal 12 will induce repelling with the negative charges positioned on 4-NP and BH_4_^−^, with the catalyst surface thus decreasing seriously the adsorption and reduction rate. Slowing down the photoreduction reaction of 4-NP at pH equal 2 compared to that at 7 is probably due to the catalyst dissolution.

This retards the well adsorption–desorption of the nitroarenes on the catalyst surface and thus affects the rate. The stability and reusability of Mn_3_O_4_/Ag_2_WO_4_ (60%) at 0.1 g amount is inspected for 4-NP (0.001 M) in 5 cycles at pH 7 without any treatment for the catalyst between the runs. The photoreduction activity decreased slightly from 100% into 84% after the fifth run indicating high efficiency and stability. This decrease in activity is probably due to accumulation of 4-aminophenol at the active sites of the Mn_3_O_4_/Ag_2_WO_4_ nanocatalyst^33^.

### H_2_ production

**Figure 12:**
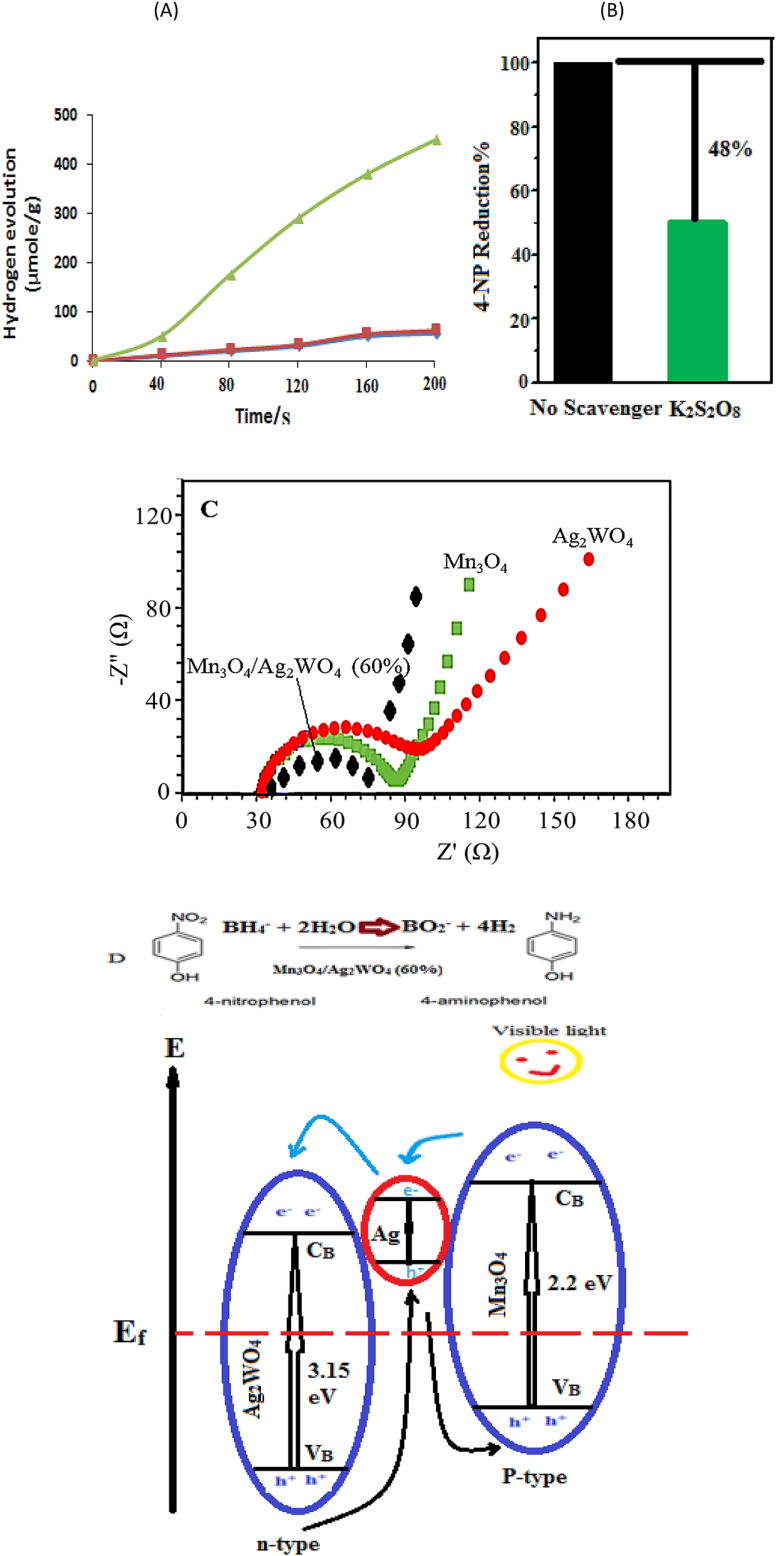
(**A**) Volume of H_2_ produced vs. time, from the borohydride reaction promoted by the different photocatalysts (1.0 g), (**B**) Effect of the K_2_S_2_O_8_ scavenger on the reduction consequences of 4-NP during the photoreduction process using Mn_3_O_4_/α-Ag_2_WO_4_ (60%). (**C**) EIS curves of the photocatalysts performed in 6.0 M KOH and (**D**) Schematic diagram for the reduction mechanism of 4-NP via electron transfer and hydrogen using the photocatalyst Mn_3_O_4_/α-Ag_2_WO_4_ (60%).

The borohydride photo-hydrolysis to signify the amount of H_2_ produced, which follows the reaction; BH_4_ (aq) + 2H_2_O (l) → BO_2_ (aq) + 4H_2_ (g) (1), was analyzed on the different catalysts to monitor the amount of generated volume of H_2_ as a function of time; which is similar to that taken place during the reduction performance of 4-NP. Figure 12a depicts the kinetic curves of the catalyst Mn_3_O_4_/Ag_2_WO_4_ (60%) in comparison with the individual analogue. Apparently, the composite catalyst showed the highest amount of H_2_ produced that comprised of 470 µ mole/g by the time of 200 s exceeding Mn_3_O_4_ and Ag_2_WO_4_ those did not exhibit notable activity (only 35 µ mole/g).

The higher activity of the former photocatalyst is probably due to increasing the electron density; as evidenced by the electronic conductivity measurement, together with the expected improvement in the electron transfer that highly enhanced following light irradiation. Figure 12b shows the trapping experiment while performing the 4-NP reduction to investigate whether electrons are involved in this reaction or not. The used K_2_S_2_O_8_ scavenger indicates a suppression of 48% manifesting that electrons are very important for the reduction process. However, the % of 52 dictates the involvement of some other species mostly as H_2_. Thus, photo-induced electrons and H_2_ of strong reductive potentials are competently reduced 4-NP.

### Mechanism for the 4-NP photocatalytic reduction

It is evident that the Mn_3_O_4_/Ag_2_WO_4_ (60%) composite has the greatest photo-current density. Because, this composite structure provides small diffusion distance for photo- motivated charges that are quickly transport into the catalyst surface. Moreover, the small electron transfer resistance of Mn_3_O_4_/Ag_2_WO_4_ (60%) signifies a high-speed interfacial charge transfer, which may be responsible for the excellent performance of 4-NP reduction. Electrochemical impedance spectroscopy (EIS) was performed to study the transfer and separation of photo-excited carriers. As confirmed in Fig. 12C, the Nyquist plot of Mn_3_O_4_/Ag_2_WO_4_ (60%) displays a much smaller semi-circle than those of Mn_3_O_4_ and Ag_2_WO_4_, reflecting a smaller charge transfer resistance for the former comparatively.

In view of above results, we suggested a mechanism of Mn_3_O_4_/Ag_2_WO_4_ (60%) in the photo-reduction of 4-NP with borohydride. The reaction of the latter with 4-NP to produce p-nitrophenolate anion is primarily attained via adsorption onto the positively charged surface of the composite. Consequently, p-nitrophenolate anions are reduced to 4-AP with borohydride. Under visible light illumination, electrons can pass from Mn_3_O_4_ into Ag_2_WO_4_ when they come into contact forming a Schottky barrier. The latter catalyst possesses a smaller work function (4.3 eV) relative to that of the former (4.5–6 eV) thus facilitating the electron transfer in the sequence from Mn_3_O_4_ to Ag_2_WO_4_. The poor photocatalytic reduction-ability for 4-NP on the individual catalysts raises the negligible importance of the bulk morphology that results in high charge recombination rate. Besides, their negatively charged surfaces rule out the nitrophenolate anions from being well adsorbed. In addition, the amorphous nature of individual catalysts as well as the Mn_3_O_4_/Ag_2_WO_4_ (40%) catalyst have shown negative effects on their activities. This is because they own less number of active coordinate, the requisite of high energy for creating e^−^–h^+^ and the high opportunity for their recombination.

Figure 12D shows the illustrated diagram of the electron transfer across the Mn_3_O_4_/Ag_2_WO_4_ interface under visible light irradiation. When they are in contact, energy bands of Mn_3_O_4_ are raised up and those of Ag_2_WO_4_ are brought down till an equilibrium position of Fermi levels is achieved. During light irradiation, electrons and holes are respectively formed in the C_B_ and V_B_ of Mn_3_O_4_.

The photo-generated electrons can simply move from the C_B_ of Mn_3_O_4_ to that of Ag_2_WO_4_ under the action of the tailored electric field where the holes remained in the V_B_ of Mn_3_O_4_. This indeed will effectively lengthen the life span of charge carriers at the nano-composite surface. The collected electrons, of strong reductive power (E_CB_ (Mn_3_O_4_) = -0.35 V vs. NHE), on the C_B_ of Mn_3_O_4_ react with nitrophenolate moieties to form 4-AP. The adsorption of both BH_4_ and 4-NP ions on the composite surface is followed by H transfer from BH_4_ to the composite forming hydridic moieties, which react with water molecules liberating H_2_ molecules that can also reduce 4-NP. The deposited Ag nanoparticles on the surface of α-Ag_2_WO_4_ can act as electron catch centers, and as a result retard the photogenerated charges recombination. Also, Ag nanoparticles can inhibit the transfer of holes from the V_B_ to the interface of the photocatalyst and solution^42^. That is why we did not detect any oxidation products for 4-NP. Simultaneously, the V_B_ potential of Mn_3_O_4_ is negative compared to the redox potential of ·OH/OH^−^ (2.38 V vs. NHE), showing that the photo-induced holes are not capable of oxidizing surface hydroxyls (and OH^−^) or H_2_O in the degradation medium to provoke the formation of •OH radicals.

To further prove the capability of the Mn_3_O_4_/α-Ag_2_WO_4_ catalyst in evoking the photo-generated charge carrier transport upon light irradiation, we traced transient photocurrent responses and linear sweep photovoltammetry measurements^43^.

**Figure 13:**
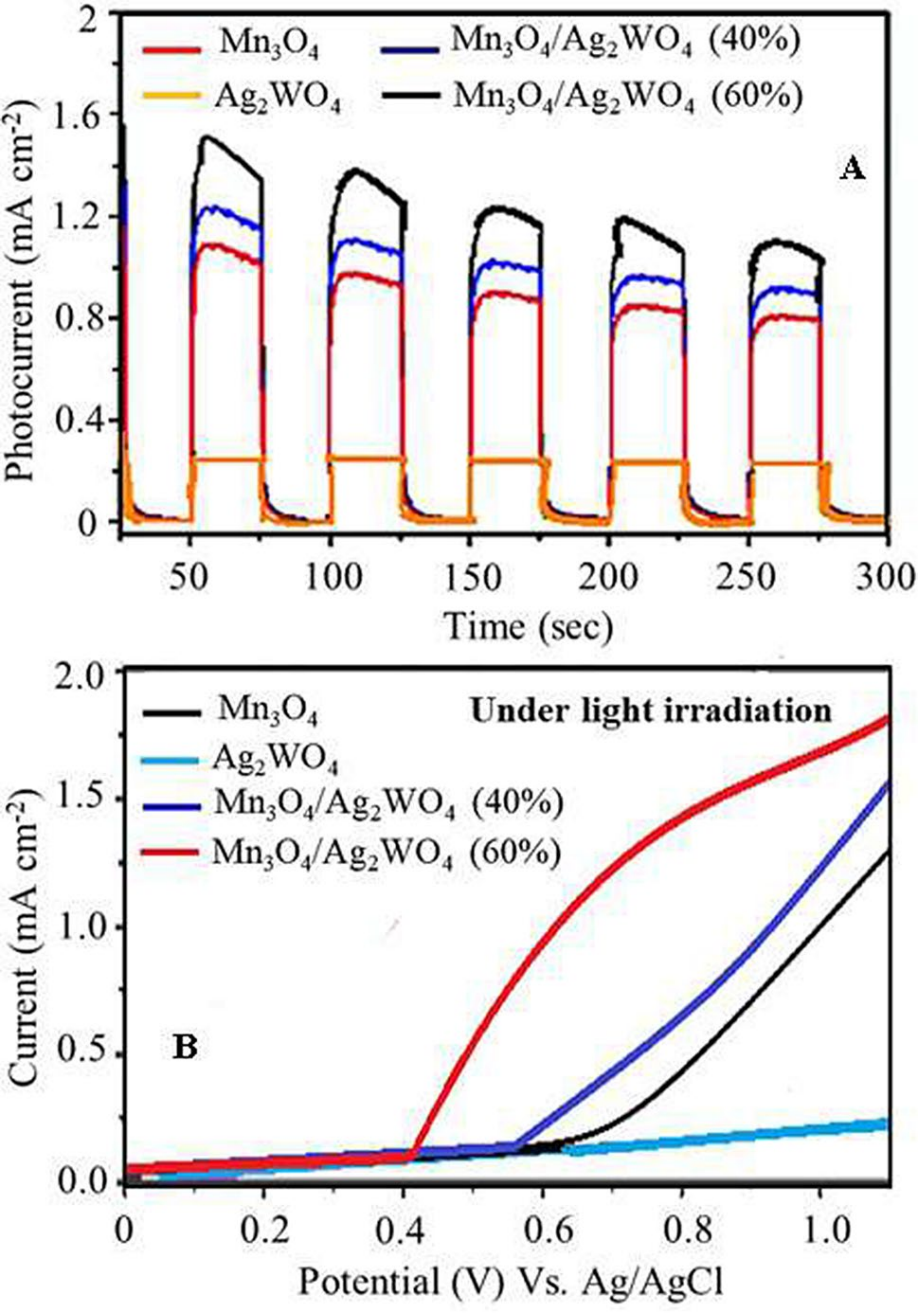
(**A**) Transient photocurrent responses and (**B**) linear sweep photovoltammetry (LSV) results of Ag_2_WO_4_, Mn_3_O_4_, Mn_3_O_4_/Ag_2_WO_4_ (40%) and Mn_3_O_4_/Ag_2_WO_4_ (60%).

Figure 13A displays photocurrent density-time curves of Ag_2_WO_4_, Mn_3_O_4_, Mn_3_O_4_/Ag_2_WO_4_ (40%) and Mn_3_O_4_/Ag_2_WO_4_ (60%) as light illuminates the catalyst surface. The photocurrent response curves are realized via using an alternating light. Apparently, the photocurrent density of Ag_2_WO_4_ was comparatively lower than that depicted for Mn_3_O_4_ that shows a decreasing trend with extending the illumination time, reaching to a value of 0.12 mA cm^−2^. Contrarily, Mn_3_O_4_/Ag_2_WO_4_ (40%) gives a transient photocurrent with a slight decrease but actually lower that that given by Mn_3_O_4_/Ag_2_WO_4_ (60%), that gives a value comparable to 0.15 mA cm^−2^. This elaborates that the latter catalyst owns stronger capability in producing as well as transporting the photo-excited charge carrier^44,45^. This is likely due to at such mentioned loading [Mn_3_O_4_/Ag_2_WO_4_ (60%)], an increase in the separation efficiency of photo-generated charge carriers (electrons-holes) is improved^46^. Figure 13B displays the plots between photocurrent density and applied voltage attained under visible light irradiation. Evidently, the photocurrent density of the catalysts rises thru the forward bias voltage, revealing a characteristic n-type semi-conductor^47,48^. It is shown that the Mn_3_O_4_/Ag_2_WO_4_ (60%) catalyst exhibits the optimal photocurrent within the same designated voltage profile, typical to the results obtained from the photocurrent density-time curves.

To understand the effect of Ag NPs on catalytic performance, a chemical excavation approach via using a mild ethanolic nitric acid (~ 10% v/v) is employed to steadily remove Ag NPs from the Mn_3_O_4_/α-Ag_2_WO_4_ (60%) surface. Accordingly, the correlated catalytic activity indicates the pertinent role of Ag NPs in the photoreduction process. This demonstrates that Mn_3_O_4_/Ag_2_WO_4_ crystal renders the aid to promote 4-NP reduction, whereas Ag NPs assist the catalyst to modulate the charge transfer. Decreasing the induction time observed in Mn_3_O_4_/α-Ag_2_WO_4_ compared with the individual analogue during the reduction of 4-NP reflects the higher diffusion of the reactants (NaBH_4_ and 4-NP) on the former surfaces. This reveals that the formed Mn_3_O_4_/α-Ag_2_WO_4_ surface owns a higher hydrophilic nature that facilitates the diffusion of the reactants as well as electrons and H_2_ and thus modulates the catalytic reduction property. Comparative investigation of catalytic performances and kinetics details with the start-of-the-art catalysts accomplished under comparable experimental conditions for 4-NP reduction are displayed in Table 2. Evidently, the Mn_3_O_4_/α-Ag_2_WO_4_ (60%) photocatalyst displays a significantly higher kinetic rate (0.067 s^−1^) than mostly used metals (Pd, Ag and Cu) in addition to some metal oxides (TiO_2_), ferrites based catalysts and Au@Ag core–shell NPs^49–57^.

**Table 2:** Catalytic reaction rates summary of 4-NP on various reported catalysts under separate conditions at the room temperature.

Catalyst name	4-nitrophenol conc. (M × 10^–3^)	Molar ratio of NaBH_4_/4-NP	Catalyst dosage (g/L)	Kinetics (s^−1^)	References
Pd − rGO	0.1	1000	1.2	4.5 × 10^−3^	^49^
N-acyl tyramine-AgNPs	1.0	15	2.5	3.89 × 10^–3^	^50,51^
Caffeic acid-AuNPs	0.4	100	~ 1.0	2.63 × 10^–3^	^52^
TiO_2_HAg2	0.18	320	1	0.025	^28^
Ag-nanoparticle/C	0.1	200	1	1.69 × 10^−3^	^53^
3D GQDs/rGO4	0.005	53	1.2	4.47 × 10^−4^	^54^
CoMn_2_O_4_	1.0	100	1.4	14.95 × 10^−3^	^55^
MnFe_2_O_4_	0.1	500	1.0	0.010	^56^
Au@Ag core–shell NPs	0.2	80	1.0	5.4 × 10^–3^	^57^
Mn_3_O_4_/Ag_2_WO_4_ (60%)	1.0	200	1.0	0.067	This work

## Conclusions

A novel Mn_3_O_4_/α-Ag_2_WO_4_ (60%) heterojunction photocatalyst fabricated by a facile sonication-deposition–precipitation route was thoroughly characterized by XRD, TEM-SAED, XPS, FTIR, UV–Vis diffuse reflectance and PL techniques. Results showed that Mn_3_O_4_/α-Ag_2_WO_4_ (60%) possessed the best photoreduction activity for nitroarenes (0.001 M); under visible irradiation, with a conversion efficiency reaching 100%, attained for example in 1 min reaction time for 4-NP with a kinetic rate equal 0.067 s^−1^. The mechanism exploration indicates that the generated hydrogen and electrons reacts with 4-NP in presence of BH_4_^−^ on the nanocomposite surface promoting the reduction process. Appropriately, the amalgamation of α-Ag_2_WO_4_ with Mn_3_O_4_ not only achieves spatial separation of photo-induced charge carriers, facilitated by the deposited Ag nanoparticles, but also boosts up the electronic conductivity. The composite Mn_3_O_4_/α-Ag_2_WO_4_ (60%) is efficiently photocatalyzed the hydrogenation of 4-NP into 4-AP (34.9 × 10^–3^ min^−1^) under mild conditions with excellent selectivity (100%) as well as it shows an improve hydrogen production under using NaBH_4_ (470 µ mole/g) delineated under the solution condition. The Mn_3_O_4_/α-Ag_2_WO_4_ (60%) photocatalyst exhibited a self-restoration ability providing a new perspective for application in the photocatalysis field**.**

## Methods

### Synthesis of Mn_3_O_4_

4.5 mmol MnCl_2_·4H_2_O and 1.0 g PVP was dissolved in 80 mL distilled water. Instantaneously, 5 mL NaOH (2.0 M) was added to the mixture, producing a light brown precipitate that refluxed under stirring for 4 h at 90 °C. The mixture was cooled down to room temperature, and the solid powder was separated by centrifugation and washed three times with distilled water and ethanol.

### Synthesis of Mn_3_O_4_/Ag_2_WO_4_-60% (60 wt. % of Ag_2_WO_4_ relative to Mn_3_O_4_)

0.20 g of Mn_3_O_4_ was dispersed into 100 mL distilled water by ultrasonic irradiation for 10 min. Then, 0.210 g of silver nitrate was added to the suspension and stirred for 60 min. An aqueous solution of sodium tungstate Na_2_WO_4_.2H_2_O; prepared by dissolving 0.213 g in 20 mL water, was drop wisely added to the previous suspension followed by refluxing at 90 °C for 60 min. The resultant suspension was then centrifuged to collect the precipitate, which washed two times with water/ethanol solution and dried in an oven at 60 °C for 24 h. The pure Ag_2_WO_4_ photocatalyst was typically fabricated as mentioned except the Mn_3_O_4_ addition. The synthesis of Mn_3_O_4_/Ag_2_WO_4_-40% was attained via using the same procedure to give 40 wt. % of Ag_2_WO_4_ relative to Mn_3_O_4_.

### Reduction of nitroarenes

To investigate the catalytic reduction of 4-nitrophenol, 4-nitro aniline and 4-nitro acetanilide, 100 mL of 0.001 M aqueous solution of the nitroaromatics was taken in a 250 mL beaker with 10 mL of 0.2 M NaBH_4_. This system was then illuminated by a visible light LED lamp of 50 W with a cut off filter (λ > 420 nm, 30 mWcm^−2^); to obviate the low emissions existed near to UV and IR margins, and fixed at a distance of 25 cm. The solution was subjected to a constant stirring. A desired amount of catalyst was added; so as to reaching 1 g/L, while stirring and continued at room temperature. The dark yellow color of the solution is progressively vanished with time, demonstrating the reduction of nitro aromatics. The reaction progress was checked via withdrawing samples from the reaction mixture at normal time intervals. The conversion of nitroaromatics to the corresponding aminoaromatics was checked by UV–Visible spectroscopy (6705 UV/Vis JENWAY). However, in the fast reduction of 4-nitrophenol, 3 mL of 0.001 M was placed in the quartz cuvette of the spectrophotometer with 3 mg catalyst and the absorbance is recorded regularly without stirring i.e. the catalyst is settle down at the cuvette bottom. The catalysts stability and reusability was examined after the reaction completion, via washing with distilled water and ethanol in sequence to remove the nitroaromatics adsorbed on the surface. The catalyst recycling test was accomplished 5 times**.**

The photocatalytic hydrogenation of nitrophenol was conducted in a 75 mL sealed glass autoclave with a quartz window for light irradiation. A typical reaction process is described as follows: 0.001 M nitrophenol and 100 mg of catalysts were dispersed in 20 mL of neat ethanol, and the suspension was then sealed in an autoclave under 0.35 MPa of H_2_ with stirring. The mentioned lamp was also employed as the light source at the same light intensity and at room temperature. After reaction, the collected products following 1.0 h reaction time were analyzed by gas chromatography-mass spectroscopy (GC–MS-Bruker, Germany) technique.

### Hydrogen generation

The catalytic hydrolysis of NaBH_4_ (200 mg in 100 mL deionized H_2_O) was carried out at ambient temperature that never exceeds 25 °C by wetting the photocatalyst in water inside a vessel system that maintained stirring at 750 rpm. The liberated H_2_ was measured using a water-displacement technique; which was completely insulated so as to guarantee that all the gas evolved are well stored, using an electronic balance with an accuracy of 0.01 g. The volume of H_2_ produced was quantified as a function of time, with repeating each experiment not less than two times to guarantee the reproducibility. The calculated relative error was no more than 2%.

### Characterization techniques

X-ray diffractions (XRD) provided with Ni-filtered copper radiation (λ = 1.5404 Å) was used to distinguish the crystal structure of the nanocomposites operated at 30 kV and 10 mA with a scanning speed of 2θ = 2.0°/min. The Fourier transform infrared (FT-IR) spectra are evaluated via a Perkin Elmer Spectrometer (RXI FT-IR), at a resolution of 1.0 cm^−1^, within the region 400–4000 cm^−1^ using the KBr technique. Diffuse Reflectance Ultraviolet–visible spectroscopy (UV–vis DRS) of the nanocomposite and the individual catalysts is measured at r.t. using UV–vis JASCO spectrophotometer (V-570) in the range of 200–800 nm. The edge energies (Eg) of allowed transitions are determined by finding the intercept of the straight line in the low-energy rise of the plot using the relation αhʋ = A(hʋ − Eg)^n^. The photoluminescence (PL) emission spectra were measured following the excitation with a continuous-wave He-Cd laser (λ = 325 nm). X-ray photoelectron spectroscopy (XPS) spectra were measured by the Thermo ESCALAB 250XI photoelectron spectroscopy system using a monochromatic Al Kα source operated at 200 W. The TEM micrographs are obtained using an FEI; model Tecnai G20, Super twin, double tilt 1010, at a pick up voltage of 100 kV. The electrochemical impedance spectroscopy (EIS) studies were made using an EG&G PAR galvanostat/potentiostat, model type 273, with an amplitude of ± 5 mV in the frequency range 10^3^–10^−2^ Hz. All photo(electro)chemical measurements of the films deposited on conducting glasses used as working electrode are conducted on an EG&G PAR potentiostat/galvanostat, model 273. A Pt electrode is used as a counter electrode and Ag/AgCl as the reference one, where the electrolyte was 0.5 M of Na_2_SO_4_ aqueous solution. The turning of the incident light on and off consecutively was permitted for a period using the 150 W halide lamp (60 mW cm^2^).

The dc electrical-resistivity is measured with an electrical circuit as illustrated elsewhere using the equation σ_dc_ = (l/A_s_). (1/R_dc_)^30,31^; where A_**s**_ is the cross-sectional area, R_dc_ is the sample resistance and l is the length of the sample.
